# Multistate models for comparing trends in hospitalizations among young adult survivors of colorectal cancer and matched controls

**DOI:** 10.1186/1472-6963-12-353

**Published:** 2012-10-09

**Authors:** Rinku Sutradhar, Shawn Forbes, David R Urbach, Lawrence Paszat, Linda Rabeneck, Nancy N Baxter

**Affiliations:** 1 Institute for Clinical Evaluative Sciences, 2075 Bayview Avenue, Toronto, ON, M4N 3M5, Canada; 2 Dalla Lana School of Public Health, University of Toronto, Toronto, Canada; 3 Department of Surgery, University of Toronto, Toronto, Canada; 4 Department of Health Policy, Management and Evaluation, University of Toronto, Toronto, Canada; 5 Toronto General Hospital, University Health Network, Toronto, Canada; 6 Department of Medicine, University of Toronto, Toronto, Canada; 7 Keenan Research Centre, Li Ka Shing Knowledge Institute, St. Micheal’s Hospital, Toronto, Canada

**Keywords:** Multistate model, Counting process, Random effects, Young adult survivors, Proportional rate regression model, Baseline rate function

## Abstract

**Background:**

Over the past years, the incidence of colorectal cancer has been increasing among young adults. A large percentage of these patients live at least 5 years after diagnosis, but it is unknown whether their rate of hospitalizations after this 5-year mark is comparable to the general population.

**Methods:**

This is a population-based cohort consisting of 917 young adult survivors diagnosed with colorectal cancer in Ontario from 1992–1999 and 4585 matched cancer-free controls. A multistate model is presented to reflect and compare trends in the hospitalization process among survivors and their matched controls.

**Results:**

Analyses under a multistate model indicate that the risk of a subsequent hospital admission increases as the number of prior hospitalizations increases. Among patients who are yet to experience a hospitalization, the rate of admission is 3.47 times higher for YAS than controls (95% CI (2.79, 4.31)). However, among patients that have experienced one and two hospitalizations, the relative rate of a subsequent admission decreases to 3.03 (95% CI (2.01, 4.56)) and 1.90 (95% CI (1.19, 3.03)), respectively.

**Conclusions:**

Young adult survivors of colorectal cancer have an increased risk of experiencing hospitalizations compared to cancer-free controls. However this relative risk decreases as the number of prior hospitalizations increases. The multistate approach is able to use information on the timing of hospitalizations and answer questions that standard Poisson and Negative Binomial models are unable to address.

## Background

The incidence of colorectal cancer (CRC) among young adults has been increasing over the past three decades. Data from the Surveillance, Epidemiology, and End Results registry indicate that the incidence of colon cancer in persons aged 20 to 40 years increased 17% between 1973 and 1999. Moreover, the incidence of rectal cancer in this age group increased 75% over this time period [1,2]. Due to improvements in disease-specific survival, a large percentage of these patients now survive 5 years or more after diagnosis of CRC [1]. However, these survivors remain at a higher risk for late effects such as late mortality and second cancers. Using the same population-based cohort discussed in this paper, a recent study by Forbes et al. (2010) found young adult survivors of CRC have a significantly higher risk of long-term death than matched controls (HR=8.2, 95% CI (5.8, 11.6)) [1].

Despite the increasing number of young adult CRC survivors long-term health effects of CRC – a disease frequently requiring multi-modal therapy including surgery, chemotherapy and irradiation – in a young population have not been well studied [1,3]. In older adults, long-term survivors of CRC are known to have an increased risk of small bowel obstruction [4,5], and treatment may result in substantial genito-urinary dysfunction [6,7]. Other disorders, including pelvic fractures [8] dementia, diabetes and osteoporosis [9], may also be associated with CRC survival. Although late effects may occur, this has not been well studied in CRC survivors, particularly in comparison to other malignancies, perhaps because of the advanced age of most patients with CRC at diagnosis. Long-term effects of CRC diagnosis and treatment may have a more substantial impact on younger survivors – younger survivors have been found to have worse quality of life and experience more role restrictions than older CRC survivors [10], and certainly young CRC survivors have a longer potential time span to experience late-effects. .The impact of CRC on hospital admissions, an indicator of significant illness, among young adult survivors compared to the general population is unknown. The risk of hospitalization over time may be greater than in younger CRC patients, however, some late effects associated with hospitalization, such as pelvic fracture after irradiation may be less common in young adults than older survivors who are at higher baseline risk. By comparing rates of hospitalization in long-term survivors and a control population we can assess long-term morbidity due to significant medical illness attributable to CRC and treatment in a group of young survivors. Additionally, higher rates of hospitalizations would imply that this population of CRC survivors has an increasing impact on the Canadian health care system and an increasing demand for hospital services [3].

Data on repeated hospitalizations over time are often referred to in the statistical literature as recurrent event data. Standard analyses are based on Poisson or negative binomial models – these approaches estimate the rate of hospitalizations by simply modeling each patient’s total number of hospitalizations over their observation period. However if one is interested in taking the timing of each hospitalization into account, then various counting process or gap time models can be adopted. In many cases, a terminal event such as death occurs which precludes the occurrence of future recurrent events. In the models mentioned above, the time of death is often treated as a censoring time, implying that patients are still at risk of experiencing further recurrent events. To overcome this issue a multistate analysis is recommended - it models the terminal event as an absorbing state, since no recurrent events can occur after this point.

Multistate models examine disease processes by describing changes in a patient’s health condition over time [11]. These models classify a patient into one of a finite number of distinct states at any given time during their follow-up [12]. Events correspond to transitions from one state to another, and the event times correspond to the transition times [13,14]. Recently, multistate models have been extended to examine recurrent event data in which a terminal event may occur [15,16]. Examples include organ transplant studies where transient graft rejection episodes are terminated by total graft rejection or death [15], and studies of cancer patients with bone metastases where the occurrence of new metastases is terminated by death [16]. Although multistate methods have been developed under such settings, the application of these models is limited in the epidemiology and clinical literature. This paper’s main objective is to study trends in hospitalizations among a cohort of young adult survivors of colorectal cancer and their matched cancer-free controls using a flexible multistate model.

## Methods

### Study population

The study consists of young adult survivors of colorectal cancer and matched cancer-free controls in Ontario, Canada. This cohort was recently studied by Forbes et al. [1] to compare long-term survival of young adult survivors and controls. Young adults have been defined by the Canadian Cancer Society of Canada as persons aged 20 to 44 years [17]. All individuals diagnosed with CRC in Ontario between January 1, 1992 and December 31, 1999, and aged 20 to 44 years at the time of diagnosis of CRC were eligible for inclusion. Diagnosis date and type of cancer diagnosis are retrieved from the Ontario Cancer Registry (OCR), a comprehensive population-based cancer registry created to capture all incident cases of cancer in the province. Patients were considered survivors if they were alive 5 years after diagnosis. Individuals were excluded if they died within 5 years of diagnosis, or if they had a diagnosis of any other cancer before their diagnosis of CRC.

Controls were identified using Ontario’s Registered Persons Database (RPDB). Five controls were randomly matched to each young adult survivor on calendar year of birth, sex, and geographic location. The referent date for a control was defined as the date of diagnosis for their corresponding matched young adult CRC survivor. Controls were only eligible for inclusion if they had no prior diagnosis of cancer before their referent date and survived a minimum of 5 years after the referent date. After the 5-year mark, survivors and controls were followed until their date of death, date of OHIP (Ontario Health Insurance Plan) eligibility loss, or until date of study end (December 31^st^, 2007).

Admissions to a hospital for acute illness are identified using the Canadian Institute for Health Information Discharge Abstract Database (CIHI-DAD). Over each individual’s follow-up period, the number of admissions and the date of each admission are recorded. Permission for data access was obtained from the Institute of Clinical Evaluative Sciences (ICES), Toronto, Ontario.

### Multistate models

Multistate models use distinct states to describe changes in a patient’s condition over time. Events correspond to transitions from one state to another, and the event times correspond to the transition times [14]. The multistate model treats death as an absorbing event, as no further admissions can occur after this point [16,18]. Note that the common survival model can be viewed as a 2-state model, where the first state represents an “alive” state and the second represents the “dead” state. Survival analysis aims to characterize the distribution of the transition time to the dead state, whereas a multistate analysis aims to describe the distribution of several transitions (not only to the dead state).

The multistate model assumes the baseline rate function is dependent on the number of prior events. A patient cannot be at risk for their k^th^ admission without experiencing admission k-1. Time *t* is measured as time in years starting from 5 years after the diagnosis date (for survivors) or from 5 years after the referent date (for controls). At any given time *t*, the multistate model allows the patients who are at risk for their 10^th^ admission, for example, to have a different baseline rate function than patients who are at risk for their 1^st^ admission. Similarly, the model assumes the baseline rate function for death varies depending on the number of admissions experienced. The model also allows for separate regression parameters to be estimated for each transition. The instantaneous transition rate [14,15,19] can be expressed as a proportional rate regression model

(1) λi,jst=λ0,jstexpxiβjs,vi.

**Figure 1:**
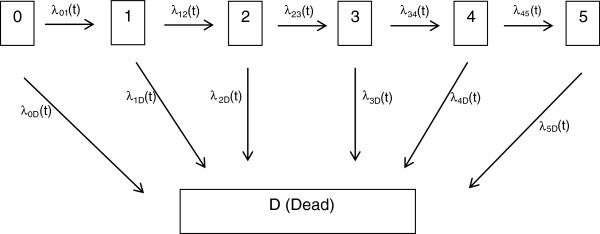
Multistate Diagram for Admissions and Death.

Function λ_i,js_(t) represents the instantaneous rate for a transition from state j to state s at time *t* for the i^th^ patient. The baseline instantaneous transition rate function λ_0, js_(t) and parameter vector **β**_js_ is specific to each j→s transition. The random effect ν_i_ accounts for the heterogeneity in the j→s transitions rates between patients [20]. Note that if we are interested in the estimate of a common regression parameter, then parameter vector **β**_js_ in the model can simply be replaced by **β**. Figure 1 provides a multistate diagram for characterizing the occurrence of hospital admissions and death. Patients in state 2, for example, are alive and have experienced two admissions; patients are in state D if they have died. From each non-absorbing state, patients can either make a forward transition to the next non-absorbing state or can make a transition to death. All models/graphs were run and created using the statistical package R [21].

The multistate methodology is custom made for prospective cohort data and it is important to be aware of methods for handling matching under such models. Cluster-specific random effects [19] can be incorporated into the multi-state model to handle correlation that may arise from matching (that is, each matched group can be considered a cluster). Our model includes patient-specific random effects, as it is important to account for variation in the transition rates between patients. In theory, one can incorporate both patient-specific and cluster-specific random effects.

## Results

This cohort study consisted of 5775 patients, among whom 917 patients were YAS of colorectal cancer and the remaining 4585 were controls. Among survivors, the mean age was 39.3 years, and the male to female ratio was 50:50. These distributions were the same in controls, as survivors and controls were matched on calendar year of birth and sex. Colon cancer was diagnosed in 642 (70.0%) young adults with CRC, and the remainder had rectal cancer. The numbers of hospital admissions for acute illness among YAS and controls are given in Table 1. Of the 917 YAS, 321 (35.0%) were admitted to a hospital at least once during their follow-up period; whereas among the 4585 controls, 889 (19.4%) were admitted at least once. The average time to the first hospitalization (from the 5-year mark) for the entire cohort is 3.06 years, which is more than double the average time from the first to second hospitalization (1.42 years). On average, the time from the second to third hospitalization is even shorter at 1.06 years. These crude numbers imply that the rate at which a hospitalization occurs increases as the number of previous hospitalizations increase.

**Table 1:** Number of Admissions to Hospital for Acute Illness Among YAS and Controls

	**Number of hospital admissions**					
	**0**	**1**	**2**	**3**	**4**	**≥ 5**
YAS (%)	596 (65.0)	157 (17.1)	68 (7.4)	34 (3.7)	18 (2.0)	44 (4.8)
Controls (%)	3696 (80.6)	573 (12.5)	175 (3.8)	67 (1.4)	32 (0.7)	53 (1.0)

**Figure 2:**
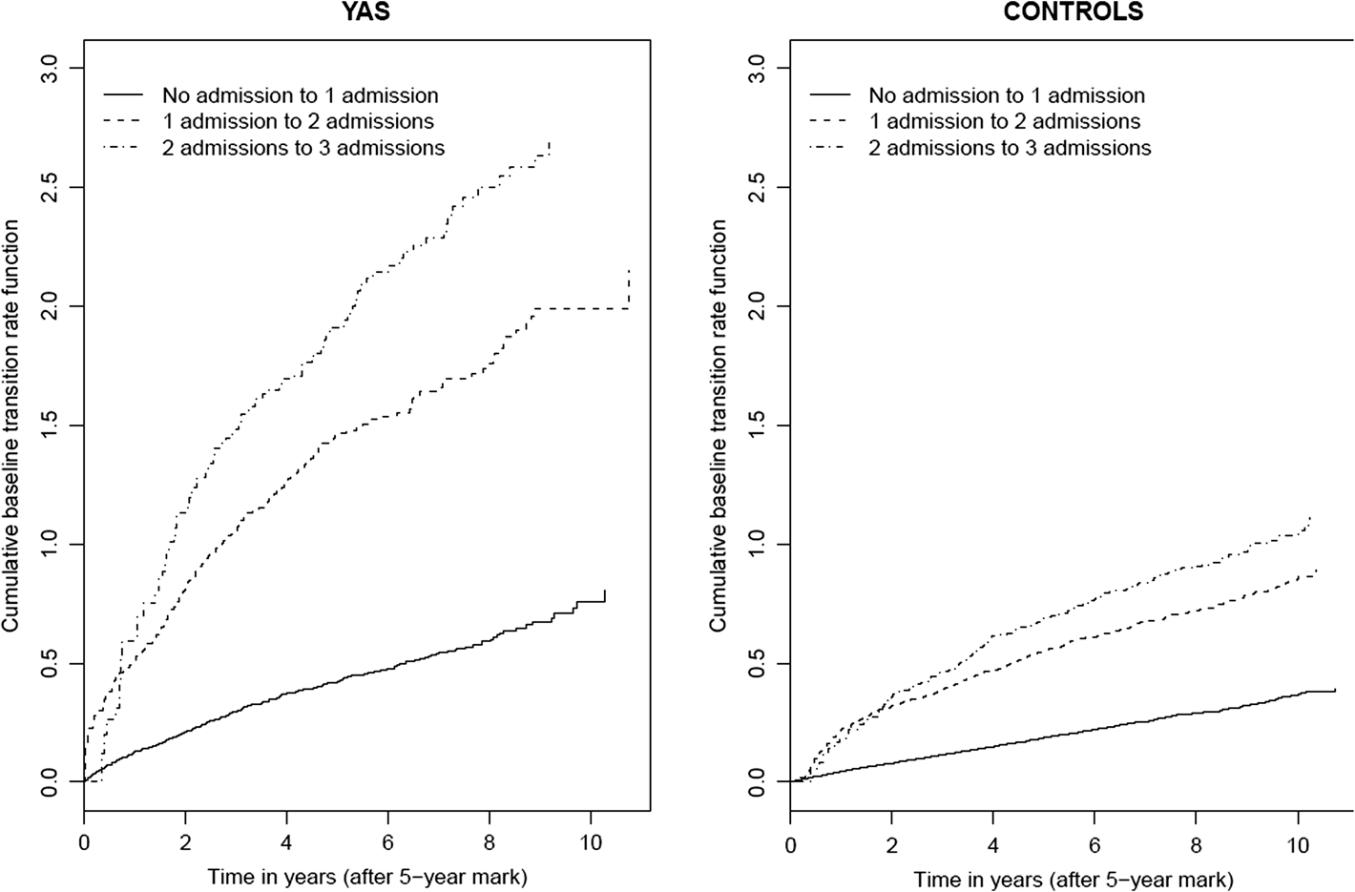
Estimates of the Cumulative Baseline Rates for Subsequent Hospital Admissions Following 0, 1, or 2, Hospital Admissions Based on the Multistate Model.

Figure 2 provides the plots of the estimated cumulative baseline rate functions for hospital admissions among survivors and controls based on the multistate model. For both groups, by examining the relative steepness of the curves, the predominant message is that at any given time t, a patient with k prior hospitalizations is at higher risk of a subsequent hospitalization than a patient with k-1 prior hospitalizations. For example, patients who have experienced 1 hospital admission (dashed line) are at higher risk of a subsequent admission than patients who have experienced no hospital admissions (solid line). Moreover, there is a slight further elevation in risk of a subsequent admission following the second hospital admission (dotted line). This justifies the use of a different baseline rate function for each admission, as adopted by multistate model. Note that the crossing of the curves in the initial stages is not concerning, as it occurs because most patients are not at risk of their 2^nd^ or 3^rd^ event, for example, for small values of t.

**Figure 3:**
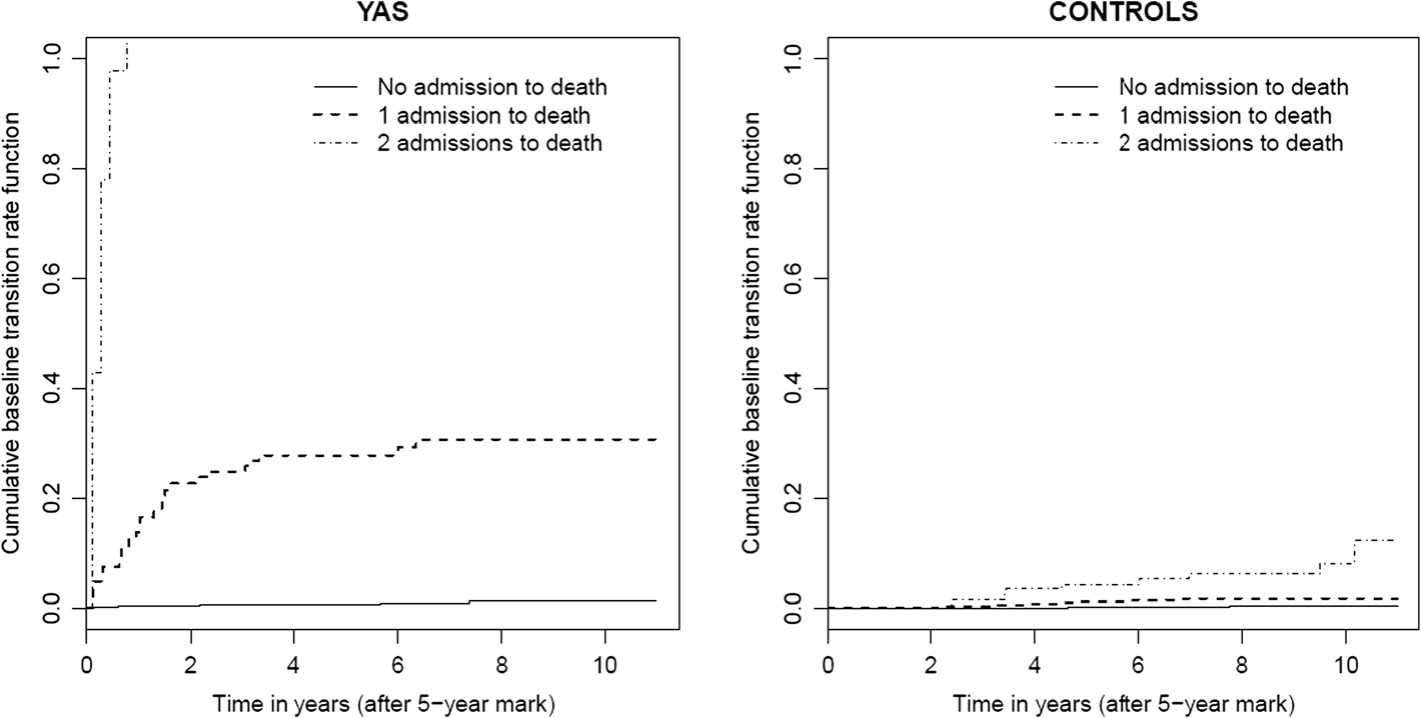
Estimates of the Cumulative Baseline Rates for Death Following 0, 1, or 2 Hospital Admissions Based on the Multistate Model.

The plots of the estimated cumulative baseline rate functions for death among survivors and controls are illustrated in Figure 3. For survivors, the functions indicate that a patient with k prior hospitalizations is at higher risk of death than a patient with k-1 prior hospitalizations. For example, survivors who have experienced 2 hospital admissions (dotted line) are at a far higher risk of death than patients who have experienced 1 hospital admission (dashed line). A similar pattern can be seen among controls, however the relative steepness of the curves is not as prominent. Although no data were dropped, the results for transitioning from 3 to 4 hospitalizations and so forth were not presented. This is because the numbers of patients experiencing these transitions during their observation periods were very small and resulted in large confidence intervals.

Table 2 presents the estimates of the relative rate of admissions comparing YAS versus controls from regression analyses based on the multistate model. The model is adjusted for income quintile, as YAS and controls are already matched by calendar year of birth, sex, and geographic location. The model also includes patient-specific random effects to handle heterogeneity. The results indicate that the rate of admissions for acute illness is much higher among YAS than controls. Among patients who are yet to experience a hospitalization, the rate of admission is 3.47 times higher for YAS than controls (95% CI (2.79, 4.31)). For those who have experienced one hospitalization, the relative rate of a subsequent admission is 3.03 (95% CI (2.01, 4.56)). Moreover, among patients that have experienced two hospitalizations, the relative rate of a subsequent admission decreases to 1.90 (95% CI (1.19, 3.03)). The notable differences in the relative rates between transitions indicate that a common regression parameter for all transitions is not appropriate. That is, using **β**_js_ in Equation [2] is more suitable than **β**. The assumption for proportional rate functions between YAS and controls is not rejected (p-value > 0.1).

**Table 2:** Results From Regression Analyses Based on the Multistate Model

	**RR for YAS vs. controls**	**95% CI**
Transition		
No admission → 1 admission	3.47	(2.79, 4.31)
1 admission → 2 admissions	3.03	(2.01, 4.56)
2 admissions → 3 admissions	1.90	(1.19, 3.03)

The model is adjusted for income quintile (YAS and controls are matched by calendar year of birth, sex, and geographic location).

## Discussion

Over the past years, the incidence of CRC has been increasing among young adults. A large percentage of these patients live at least 5 years after diagnosis, but it is of question whether their rate of hospital admissions after this 5-year mark is comparable to the general population. Additionally, identifying an increased risk of hospitalization in these long-term survivors would indicate the persistence of late-effects of diagnosis and treatment. To the authors’ knowledge, this is the first population-based study comparing the rate of hospital admissions specifically among young adult survivors of CRC and matched cancer-free controls. We found that even more than 5-years after diagnosis and treatment, young CRC survivors have a persistently higher risk of hospitalization over time. The difference between survivors and controls is greatest for those who have not experienced a hospitalization yet. And even in survivors and controls experiencing multiple admissions, indicating the presence of significant medical illnesses in both groups, CRC survivors are still more likely to have a subsequent admission. This indicates that young CRC survivors have an increased burden of illness even compared to controls with significant medical illnesses. Understanding hospitalization patterns in this cohort can help determine the impact of this population on the Canadian health care system. For example, awareness of whether the relative rate of hospitalizations varies based on the number of previous hospitalizations can assist in establishing the need for hospital services and the provision of timely hospital care. Due to high costs associated with hospital services, this topic has received considerable attention among health care professionals, policy makers, health care system administrators, and of course the general public [1].

Data on repeated hospitalizations in which death is a terminal event are often simply viewed as count data, where the endpoint for each patient is the number of events experienced over their period of observation. The Poisson model is commonly used to analyze count data, however its distributional assumptions cannot handle over-dispersion that is typically exhibited by hospital admission data. The negative binomial model [22,23], which is derived as a Poisson-gamma mixture, can accommodate over-dispersion but still views the event data on each patient as a count. To incorporate the time of each event into the analysis, an extension of the Cox proportional hazards model known as the Andersen-Gill counting process model [24,25] can be implemented. Although this model allows the event rate to change over time, it assumes that the baseline rate function is not dependent on the number of prior events. In addition, death is treated in the same way that end-of-study or loss to follow-up is treated - that is, patients are simply right-censored at the time of death. The Andersen-Gill model is appropriate to implement for our data as long as it is supplemented by also modeling the hazard of death.

The multistate model treats death as an absorbing state and is able to estimate the rate of transition to death from each non-absorbing state. The multistate model allows the rate of admissions to change over time. It provides an admission-specific estimate for the baseline rate function, which is necessary as shown in Figure 2. It also allows one to determine if the relative rate of admissions changes based on the number of prior hospitalizations. If the point estimate of the relative rate for each transition in the multistate model were similar, and if the estimates of the baseline rate functions for each transition were also similar, then this may warrant use of simpler models.

## Conclusion

In summary, among both young adult survivors of colorectal cancer and controls, the risk of a subsequent hospital admission increases as the number of prior hospitalizations increases. While the relative difference in the rate functions for hospital admissions between survivors and controls decreases as the number of prior hospitalizations increases, CRC survivors still experience a higher rate of subsequent admissions. In addition, the risk of death among survivors and controls increases as the number of prior hospitalizations increases. This increase is substantial among survivors that have experienced more than 1 hospitalization. These findings indicate even in long-term survivors, young adults continue to experience substantial morbidity from CRC diagnosis and treatment. Ongoing survivorship care planning, beyond the usual time period for cancer surveillance, may be useful in this group to attempt to mitigate the impact of the disease on long-term outcomes. This is exploratory research and further studies examining risk factors for admissions in this group are needed to find specific interventions to help reduce the long-term burden of disease.

Inclusion into the cohort requires all young adult survivors to be living 5 years from diagnosis. Patients that experience recurrent disease within the 5-year window but are still alive are not excluded from the study. Since disease status can affect the patient risk of hospital admission and death [1], it is of interest to implement the multi-state models for a recurrence-free cohort. Although there is no approach to directly identify recurrent disease using available administrative or cancer registry data, an algorithm has been developed by Tan [26] and implemented by Forbes et al. [1] that distinguishes patients with recurrent disease from those who were diseas-free 5 years after diagnosis. Applying the multi-state models to a recurrence-free cohort requires further investigation and is of current interest.

## Competing interests

The authors declare that they have no competing interests.

## Authors’ contribution

RS designed and implemented the study, collected, analyzed and interpreted data, and wrote and completed the manuscript. SB contributed to the design and implementation of the study, collection of the data, and to the writing and completion of the manuscript. DRU contributed to the interpretation of data, and to the writing and completion of the manuscript. LP contributed to the interpretation of data, and to the writing and completion of the manuscript. LR contributed to the interpretation of data, and to the writing and completion of the manuscript. NNB supervised and contributed to the design and implementation the study, collection and interpretation of the data, and to the writing and completion of the manuscript. All authors read and approved the final manuscript.

## Funding

This work was supported by a Canadian Institutes of Health Research Operating Grant. Dr. Forbes was supported by an American Society of Colon and Rectal Surgeons, General Surgery Resident Research Initiation Grant. Dr. Paszat is supported by a clinician scientist salary from the Ministry of Health and Long-term Care of Ontario. Dr. Baxter holds the Cancer Care Ontario Health Services Research Chair and an Early Researcher Award from the Ontario Ministry of Research and Innovation. The funding sources had no role in the design, conduct, or reporting of this study, or in the decision to submit the manuscript for publication. This study was conducted at the Institute for Clinical Evaluative Sciences (ICES), which is funded by an annual grant from the Ontario Ministry of Health and Long-Term Care (MOHLTC). The opinions, results and conclusions reported in this paper are those of the authors and are independent from the funding sources. No endorsement by ICES or the Ontario MOHLTC is intended or should be inferred.

## Pre-publication history

The pre-publication history for this paper can be accessed here:

http://www.biomedcentral.com/1472-6963/12/353/prepub

## References

[B1] Forbes SS Sutradhar R Paszat LF Rabeneck L Urbach DR Baxter NN Long-term survival in young adults with colorectal cancer: a population-based study Dis Colon Rectum 2010 53 973 978 10.1007/DCR.0b013e3181cf8341 20551747

[B2] O’Connell JB Maggard MA Liu JH Etzioni DA Livingston EH Ko CY Rates of colon and rectal cancers are increasing in young adults Am Surg 2003 69 866 872 14570365

[B3] Bradley NME Lorenzi MF Abanto Z Hospitalizations 1998–2000 in a British Colombia population-based cohort of young cancer survivors: report of the childhood/ adolescent/ young adult cancer survivors (CAYACS) research program Eur J Cancer 2010 46 2441 2448 10.1016/j.ejca.2010.05.001 20732288

[B4] Baxter NN Hartman LK Tepper JE Ricciardi R Durham SB Virnig BA Postoperative irradiation for rectal cancer increases the risk of small bowel obstruction after surgery Ann Surg 2007 245 553 559 10.1097/01.sla.0000250432.35369.65 17414603 PMC1877029

[B5] Birgisson H Påhlman L Gunnarsson U Glimelius B Late gastrointestinal disorders after rectal surgery with and without preoperative radiation therapy Br J Surg 2008 95 206 213 17849380 10.1002/bjs.5918

[B6] Den Oudsten BL Traa MJ Thong MS Martijn H De Hingh IH Bosscha K Van de Poll Franse LV Higher prevalence of sexual dysfunction in colon and rectal cancer survivors compared with the normative population: a population-based study Eur J Cancer 2012 Epub ahead of print 10.1016/j.ejca.2012.04.004 22608772

[B7] Pollack J Holm T Cedermark B Altman D Holmström B Glimelius B Mellgren A Late adverse effects of short-course preoperative radiotherapy in rectal cancer Br J Surg 2006 93 1519 1525 10.1002/bjs.5525 17054311

[B8] Baxter NN Habermann EB Tepper JE Durham SB Virnig BA Risk of pelvic fractures in older women following pelvic irradiation. JAMA 2005 294 2587 2593 10.1001/jama.294.20.2587 16304072

[B9] Khan NF Mant D Carpenter L Forman D Rose PW Long-term health outcomes in a British cohort of breast, colorectal and prostate cancer survivors: a database study Br J Cancer 2011 105 Suppl 1 S29 S37 10.1038/bjc.2011.420 22048030 PMC3251947

[B10] Jansen L Herrmann A Stegmaier C Singer S Brenner H Arndt V Health-related quality of life during the 10 years after diagnosis of colorectal cancer: a population-based study J Clin Oncol 2011 29 3263 3269 10.1200/JCO.2010.31.4013 21768465

[B11] Andersen PK Keiding N Multi-state models for event history analysis Stat Methods Med Res 2002 11 91 115 10.1191/0962280202SM276ra 12040698

[B12] Sutradhar R Barbera L Seow H Howell D Husain A Dudgeon D Multistate analysis of interval-censored longitudinal data: application to a cohort study on performance status among patients diagnosed with cancer Am J Epidemiol 2011 173 468 475 10.1093/aje/kwq384 21193535

[B13] Andersen PK Green A Robustness to differential mortality of incidence estimation in an illness-death-emigration model Scand J Stat 1985 12 63 68

[B14] Kalbfleisch JD Prentice RL The statistical analysis of failure time data 2002 2 New York, NY: John Wiley & Sons, Inc

[B15] Cook RJ Lawless JF The statistical analysis of recurrent events 2007 New York, NY: Springer

[B16] Cook RJ Major P Multistate analysis of skeletal events in patients with bone metastases Clin Cancer Res 2006 12 20 Suppl 6264s 6269s 17062711 10.1158/1078-0432.CCR-06-0654

[B17] Cancer Care Ontario Cancer in Young Adults in Canada 2006 Toronto, Canada, 2006 ISBN 0-921325-10-X (print), ISBN 0-921325-11-8 (pdf)

[B18] Beyersmann J Allignol A Schumacher M Competing risks and multistate models with R 2012 New York: Springer

[B19] Sutradhar R Cook RJ Clustered progressive multi-state processes under incomplete observation: application to joint damage in psoriatic arthritis J R Stat Soc Ser C 2008 57 553 566 10.1111/j.1467-9876.2008.00630.x

[B20] Putter H Van Houwelingen HC Frailties in multi-state models: Are they identifiable? Do we need them? Stat Methods Med Res 2011 Nov 23 Epub ahead of print 10.1177/0962280211424665 22116343

[B21] R Development Core Team R: a language and environment for statistical computing 2009 Vienna, Austria: R Foundation for Statistical Computing Available from: http://www.R-project.org. Accessed April 6, 2011

[B22] Dobson AJ Barnett AG An introduction to generalized linear models 2008 3 Taylor and Francis Group: Chapman and Hall/CRC

[B23] Cameron AC Trivedi PK Regression analysis of count data 1998 Cambridge, UK: Cambridge University Press

[B24] Andersen PK Gill RD Cox’s regression model for counting processes: a large sample study Ann Stat 1982 10 1100 1120 10.1214/aos/1176345976

[B25] Therneau TM Grambsch PM Modeling survival data: extending the Cox model 2000 New York, NY: Springer Publishing Company

[B26] Tan J The processes of care after colorectal cancer surgery in Ontario [master’s thesis] 2008 Toronto, Ontario: University of Toronto 93

